# Newborn Hearing Screening and Early Diagnostic in the NICU

**DOI:** 10.1155/2014/845308

**Published:** 2014-06-09

**Authors:** Maria Francisca Colella-Santos, Thaís Antonelli Diniz Hein, Gabriele Libano de Souza, Maria Isabel Ramos do Amaral, Raquel Leme Casali

**Affiliations:** ^1^The Department of Development Human and Rehabilitation (CEPRE), Faculty of Medical Sciences, State University of Campinas (FCM/UNICAMP), Rua Tessália Vieira de Camargo, 126 Caixa Postal 6111, Cidade Universitária “Zeferino Vaz,” 13083-887 Campinas, SP, Brazil; ^2^Child and Adolescent Health Program, Center for Investigation in Pediatrics, Faculty of Medical Sciences, State University of Campinas (FCM/UNICAMP), Rua Tessália Vieira de Camargo, 126 Caixa Postal 6111, 13083-887 Campinas, SP, Brazil

## Abstract

The aim was to describe the outcome of neonatal hearing screening (NHS) and audiological diagnosis in neonates in the NICU. The sample was divided into Group I: neonates who underwent NHS in one step and Group II: neonates who underwent a test and retest NHS. NHS procedure was automated auditory brainstem response. NHS was performed in 82.1% of surviving neonates. For GI, referral rate was 18.6% and false-positive was 62.2% (normal hearing in the diagnostic stage). In GII, with retest, referral rate dropped to 4.1% and false-positive to 12.5%. Sensorineural hearing loss was found in 13.2% of infants and conductive in 26.4% of cases. There was one case of auditory neuropathy spectrum (1.9%). Dropout rate in whole process was 21.7% for GI and 24.03% for GII. We concluded that it was not possible to perform universal NHS in the studied sample or, in many cases, to apply it within the first month of life. Retest reduced failure and false-positive rate and did not increase evasion, indicating that it is a recommendable step in NHS programs in the NICU. The incidence of hearing loss was 2.9%, considering sensorineural hearing loss (0.91%), conductive (1.83%) and auditory neuropathy spectrum (0.19%).

## 1. Introduction

Neonatal intensive care units (NICU) have experienced great development in the last 20 years. The mortality rate of high-risk newborn infants has gradually decreased as medical science has advanced. In Brazil, recent data revealed increased incidence of prematurity and low birth weight. One of the most important health indicators that showed improvement was the reduction in infant mortality rate [1]. Consequently, the probability of survival began to reach significant portions of about three million children born every year in the country [1].

It is known that the same conditions responsible for the deaths during the neonatal period are the most important causes of illness of the neonates. Neonatal morbidity associated with particularly severe asphyxia, severe infection, congenital anomalies, and severe respiratory distress results in delayed death or serious sequels [1]. Newborns who resist neonatal complications become prone to manifest deviations in development, including peripheral and/or central hearing impairment. The incidence of bilateral hearing loss in this population is estimated at two to five of every 100 newborns, much higher than that of the low-risk population whose prevalence is 1 to 3/1000 [2].

Many studies have reported that damage caused by hearing loss in infancy is often irreversible, affecting not only the development of speech and language but also the cognitive, intellectual, cultural, and social child development [3]. Early acoustic stimulation, especially at six months of age, leads to increased nerve connections and consequently better rehabilitation of auditory pathways [4].

Universal neonatal hearing screening (UNHS) is the first step in a neonatal hearing health program. It must be followed by multidisciplinary care for diagnosis and should be started as an early intervention process with the use of personal amplification devices and communication habilitation.

Risk factors associated with hearing loss were recommended by the Joint Committee on Infant Hearing (JCIH) in 2007 [5]. The use of risk factors is no longer recommended to select children who should undergo hearing screening. Studies have shown that only 50% of the pediatric population with congenital hearing loss would be identified by this procedure. However, it is essential to identify risk factors for hearing loss, because an infant with any of these factors in neonatal history has a greater chance of experiencing hearing loss. Additionally, it can guide the approach to be adopted after the results of the hearing screening [6].

JCIH also recommended the use of physiological procedures for screening (EOAE, evoked otoacoustic emissions, and AABR, automated auditory brainstem response). For neonates without risk factor for hearing loss (RFHL), any of the methods are considered appropriate. However, for neonates with RFHL and especially those who remained in the NICU, the use of AABR is indicated, considering the higher occurrence of retrocochlear losses, such as the auditory neuropathy spectrum disorder that cannot be identified when using EOAE.

For the diagnosis of hearing loss, a protocol with electrophysiologic measures, EOAE, tympanometry, and acoustic reflex testing is recommended. This battery of tests is critical to determine the type, degree, and configuration of hearing loss and to guide auditory rehabilitation [7].

The Woman's University Hospital (CAISM) at State University of Campinas (UNICAMP) is a teaching hospital that is considered the largest hospital of attention to women's health in the state of São Paulo, Brazil. The CAISM is prepared to receive high-risk pregnancies by maternal and/or fetal diseases of an extensive region of São Paulo totaling more than 60 counties. The hearing screening procedures performed at this hospital have been modified over the years. From the start, the hearing screening program for newborns admitted to the NICU used AABR, whenever it was possible, prior to discharge, in a single step. In 2011, new equipment was acquired and in 2012 the retest was introduced. Currently, it is important to evaluate the results obtained by the program in order to support its implementation. Thus, the objective of this research was to evaluate the outcome of the results in neonatal hearing screening program and audiological diagnosis in neonates who were hospitalized in the neonatal intensive care unit.

## 2. Methods

### 2.1. Study Design

This is a clinical, prospective, cross-sectional study. The institutional review board approved this study (Protocol no. 1085/2009).

The inclusion criteria were neonates who remained in the NICU from CAISM/Unicamp for at least 48 hours, from March 2011 to April 2013.

The exclusion criteria were neonates who stayed less than 48 hours in the NICU and those with defects of auricles, which prevented the performance of AABR, and infants born in another hospital of the city, who died or who have not completed all stages of the study.

### 2.2. Study Subjects

The sample was divided into two groups according to the date of birth of neonates and hearing screening procedures:

*Group I (GI):* neonates born between March 2011 and March 2012 who underwent NHS in one stage;

*Group II (GII):* neonates born between April 2012 and March 2013, who underwent two-stage NHS (test and retest).

### 2.3. The Study Procedures

Initially, doctors of the NICU team selected newborns that could complete the hearing screening on the days when the screening was performed, considering the general conditions of the neonate and the probable date of hospital discharge. Then, identification data, birth conditions, and risk factors for hearing loss present in clinical history were collected from medical records and/or discharge summary of the child (Box 1).

Newborns were screened by an audiologist, using AABR (Accuscreen, GN Resound) with click-type stimulus on the intensity of 35 dB NA, preferably before hospital discharge. When it was not possible, the doctor responsible for the discharge scheduled the screening.

AABR was accomplished by placing electrodes, according to the equipment manual, with white on the top of the forehead, black on the cheek, and red on the nape of the neck. An ear probe tip was used in one ear at a time, randomly, according to the position of the neonate in the cradle or with the mother in natural sleep. The test result was recorded in the book of vaccination and/or the newborn medical record. Infant passed the NHS when responding to clicking the 35 dB bilaterally. In this case, the neonate was discharged or referred for follow-up of auditory and language development at 6 months when presenting risk factors associated with delayed-onset, or progressive hearing loss.

In case of the referred result, the procedures were different for groups GI and GII.

*Group I:* infants were directly referred for complete audiological evaluation.

*Group II:* infants underwent retest with the same initial screening procedure (AABR), performed in the hospital, by appointment, approximately one month after discharge. Newborns who passed the retest were referred for follow-up of auditory and language development. Those who failed retest were referred for complete audiological evaluation.

The audiological evaluation was performed at the Laboratory of Diagnostic Audiology Assessment of Children at Unicamp. The child remained in natural sleep and the procedures were as follows:

- anamnesis with parents and otoscopic exam;
- assessment of the middle ear tympanometry and the ipsilateral acoustic reflex at 500 to 4000 Hz, with a 1000 Hz probe tone in infants aged 0 to 6 months and with a 226 Hz in infants older than 6 months, conducted through a Middle Ear Analyzer-Impedance Audiometer, AT235h, Interacoustics;
- transient evoked otoacoustic emission (TOAE) captured by 292 ILO USBII equipment;
- evoked auditory brainstem response (ABR), electrophysiological threshold and integrity of the auditory pathway conducted through Eclipse EP 25, Interacoustics with insert earphones. The skin was cleaned with alcohol and an abrasive paste before applying the conducting gel. Surface electrodes were the active electrode (Fz) and ground (Fpz) on the forehead, and the reference electrodes on the right (M2) and left (M1) mastoids. Impedance between electrodes was less than 3 KOhms, as recommended by the manufacturer. A nonvariable intensity of 80 dBHL was used to assess the integrity of auditory pathways from the inner ear to the brainstem and allowed the identification of possible changes in this path. The parameters analyzed were absolute latencies of waves I, III, and V; latency and interpeaks I-III, III-V, and I-V; wave V amplitude compared to the amplitude of wave I; interaural difference in interpeak I-V or latency of wave V. The electrophysiological threshold was obtained by the stimulus presented at decreasing intensities, until the lowest intensity that triggered the onset of wave V for click (2000 a 4000 Hz) and 500 and 1000 Hz tone bursts. Stimulation was repeated twice to check the reproducibility of track and ensure the presence of response.

It was considered normal hearing when the infant had lower electrophysiological thresholds/equal to 30 dBHL, absolute and interpeak within the expected values for gestational age latencies, and responses as expected in other procedures.

Cases with abnormal results were referred to ENT assessment in the Clinical Hospital of Unicamp and the children were subjected to physical and/or imaging examination by an otolaryngologist physician (ENT) team.

The child's hearing was classified as normal or hearing loss from the joint analysis of the outcome of audiological and ENT evaluation. The diagnosis of conductive hearing loss occurred when the otorhinolaryngological evaluation revealed disorders of the middle ear by otoscopy associated with increased values of waves I, III, and V absolute latencies and normal I-III, II-V, and I-V interpeaks, plus no transient or distortion product otoacoustic emissions, tympanogram type B or C, and no acoustic reflex. Sensorineural hearing loss was diagnosed when waves I, III, and V absolute latencies and I-III, III-V, and I-V interpeaks were normal or there was absence of wave I and prolongation of latencies of waves III and V or total absence of waves, depending on the degree of the hearing loss. Moreover, this diagnostic included electrophysiological thresholds greater than 30 dB, no TOAE, tympanometry type A, and presence or absence of acoustic reflexes, according to the degree of loss. The auditory neuropathy spectrum was characterized by absence of responses on ABR at the maximum intensity of the device (100 dB), presence of cochlear microphonic, presence of TOAE, tympanogram type A, and no ipsilateral acoustic reflexes.

All data collected in the child's records, as well as the results obtained by the hearing screening and other assessments performed, were recorded in a computerized database. Tables of the results were constructed. To compare groups I and II regarding qualitative characteristics, Chi-square test or Fisher exact test was applied. The same tests were used to study the outcome of the hearing screening and audiological diagnosis for GI and GII. To assess the risk of hearing loss according to the presence of risk factors, the prevalence ratio and its confidence interval were calculated. The significance level was 5%; data in which statistically significant differences were found are highlighted in bold. The SAS software version 9.2 was used for this analysis.

## 3. Results

The study sample consisted of 929 live newborns, 52.5% male, 66.1% preterm births, and 42.7% underweight (weight between 1500 and 2500 g).

The GI consisted of 488 newborns (NB) and GII consisted of 441 NB. There were no statistical difference between GI and GII and the variables gender (*P* = 0.44), gestational age (*P* = 0.91), and birth weight (*P* = 0.85), which gave the group homogeneity.

**Figure 1:**
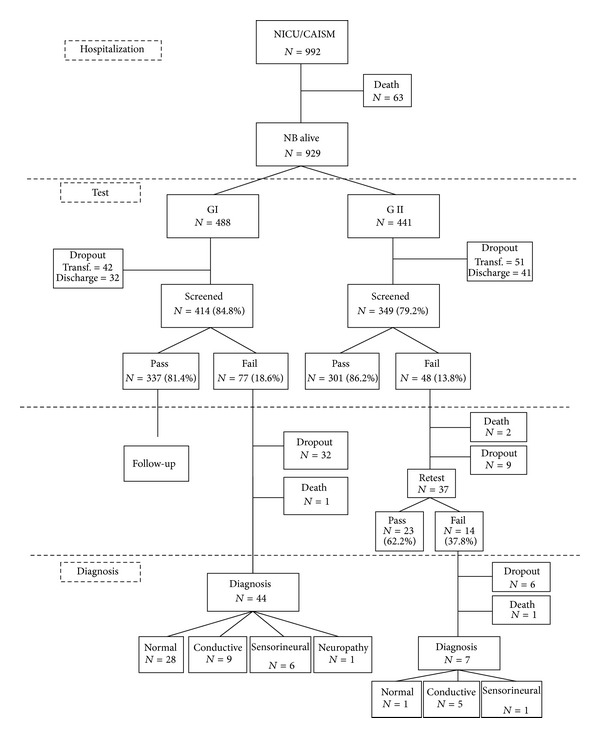
Flowchart of NHS test and retest and audiological and ENT diagnosis.

Figure 1 presents the NHS procedure (test and retest, audiological and ENT diagnosis) in GI and GII.

Table 1 shows a comparison of the results obtained in the NHS for GI-test and GII-test and retest.

Table 2 presents the results related to ENT and audiological diagnosis, for GI and GII. The incidence of hearing loss can be observed in Table 3.

In Table 4, we studied the period the NHS performed on days after birth and postconceptual age in weeks, as well as the relationship between the outcome of the NHS and the period in which it was performed.

Table 5 shows evasion in hearing screening and audiological diagnostics processes for studied groups.

Table 6 presents the frequency of risk factors of infants who underwent hearing screening, step test (*n* = 763).

It was found that the risk factor related to craniofacial anomalies involving the ear and temporal bone significantly influenced the outcome of the NHS test (*P* < 0.0001), retest (*P* < 0.0078), and the audiological diagnosis (*P* < 0.0431). Neonates with this indicator showed a greater number of failures in hearing screening test and retest, and also in the diagnosis of hearing loss compared to normal hearing and conductive loss compared to sensorineural.

The risk factors for birth weight less than 1500 g, prematurity, neonatal and congenital infections, and anoxia were studied linked because they are frequent in newborns from NICU. It was verified that they did not affect the outcome of the NHS (*P* = 0.0598). In this case, the diagnosis of normal hearing was more frequent when compared with the diagnosis of hearing loss (*P* = 0.0236). The statistical difference also occurred when comparing the type of hearing loss, with the most common being the conductive type (*P* = 0.0425).

The results showed higher failure rate in neonates with genetic syndromes that usually express hearing loss, both in GI (*P* < 0.0001) and in GII in the retest (*P* = 0.0011).

The only neonate who presented the diagnosis of neonatal asphyxia showed sensorineural hearing loss.

## 4. Discussion

Neonatal hearing screening is the main mode for early detection of hearing loss. The procedure should be fast and simple and select those most likely to have an alteration in auditory function [6]. The use of AABR is recommended as a screening procedure, which evaluates the auditory system up to the brainstem structures, and is practical and easy to apply [5]. The equipment uses clicks stimulus, does not allow visualization of the waves, and automatically sends PASS or REFER response. It is a method with high sensitivity (ability of the test to identify hearing loss) and specificity (ability to identify the individual listener as normal), Hall et al. [8]. Some studies indicate median specificity by considering the rare upward losses that are not identified by the method, besides conductive loss.

Hearing screening was performed in 82.1% of 929 live births that remained in the NICU for more than 48 hours (Figure 1). We intended to perform universal hearing screening as recommended by JCIH [5]. However, some factors made it impossible to reach the index of greater than or equal to 95% of children screened. Due to a large demand for admissions in the NICU from CAISM, newborns in better condition but not eligible for hospital discharge yet are often transferred to other health services. This particularity of service prevented the hearing screening before hospital discharge in all cases transferred due to interference of the equipment that the child still had to use in the hearing screening procedure. Furthermore, in many cases, the screening was not recommended because the newborn was taking ototoxic medications or other treatments that should be kept after the transfer and that could affect the auditory system and therefore modify the results of the hearing screening. These cases were scheduled for further hearing screening in CAISM, since many hospitals that receive transferred neonates lack the hearing screening service. However, many of these cases did not show up, even after a second contact. Frequent absences also occurred in cases of neonates who were discharged mainly on weekends, when the screening service was not available. Associated with these factors, the family's lack of awareness about the importance of returning for hearing screening and lack of resources for going to the service, among other factors, could also have contributed to the absences.

Several international studies have reached index of coverage greater than or equal to 95% and were the ones who conducted the hearing screening especially before hospital discharge [9–12]. Brazilian studies showed coverage rates between 69% and 90% [13, 14]. Some studies compared the results in NHS when it was performed before hospital discharge or after discharge (immunization clinics, e.g.) and showed coverage of approximately 88% [15, 16]. In this study, the coverage was similar to research reports that also had to schedule the NHS, because it was not possible to screen all neonates before hospital discharge.

On the global scene, the UNHS was recommended by the United States Preventive Services Task Force [17] and European Consensus Statement on Neonatal Hearing Screening [15] in 2008. In several countries, NHS is widespread, while in others it is considered a very expensive process and its value is questionable [16].

In developing countries, the challenges are greater. The high birth rate associated with the high prevalence of hearing loss combined with frequent exposure to risk factors makes the coverage of UNHS more difficult [18]. In addition, there is a poor health system parallel to insufficient funding. Many countries have already recognized the importance of early detection of hearing loss, but there was not a chance to make it universal in national terms yet. Generally the screening occurs in isolated services with varying results.

Thus, to increase the number of screened newborns and achieve coverage greater than 95% it is recommended, whenever possible, that NHS be performed prior to hospital discharge, which minimizes missing schedules and hence increases the quality of care and cost benefit of the program. Another factor that may improve adherence to NHS is the guidance to health professionals and the families of newborns, focusing on the importance of hearing for the development of speech and language, the explanations about the NHS, its role in early detection of hearing loss, and the importance of attending the service to perform the scheduled date of the hearing test.

Most children who performed hearing screening resulted in passing bilaterally, as the observed response to clicks on the AABR was found at 35 dB (Figure 1).

When comparing the results obtained in the NHS between GI and GII, we found a significant difference between the results. In GII-test and retest, there was a 4.1% rate of referral, whereas in the GI-test, the referral occurred in 18.6% of infants (Table 1). Thus, the retest decreased to 4% refer rate, as recommended by the committees and reduced the dropout rate for the diagnosis. Consequently, it was possible to reduce unnecessary referrals and minimize stress, distress and parental anxiety related to income hearing screening. The reduced rates obtained in the literature are based on two stages of NHS-test and retest [19, 20]. There are studies that performed more than one testing before hospital discharge, which further reduced failure rates [21].

In our research, infants who failed underwent the step of diagnosis. In GI, otorhinolaryngological and audiological diagnostics showed that of all children who completed the procedure, 62.2% (28 cases) presented normal hearing. Hearing loss occurred in 35.5% (16) of infants of which 20.0% (9 cases) were conductive, 13.6% (6 cases) sensorineural, and 2.2% (1 case) with auditory neuropathy spectrum disorder (Figure 1). In GI, there was a high rate of false positives, because most infants who underwent diagnosis evaluation presented normal results.

In GII, normal hearing occurred in 12.5% of cases. The hearing loss occurred in 75% of children, of which 62.5% were conductive and 12.5% sensorineural. Comparing these results with the groups, it was found that there was a significant difference (Table 2). In GII, there were fewer infants with normal hearing and greater relative number of infants with hearing loss than the GI. The retest decreased the false positive in GII and most children who failed the retest presented hearing loss.

The incidence of hearing loss in the sample was approximately 3%, of which 0.91% were sensorineural, 0.13% with auditory neuropathy spectrum disorder (ANSD), and 1.8% conductive (Table 3). The high incidence in the literature refers to sensorineural hearing loss normally. In neonates that remained in NICU, values ranged from 0.8% to 4.9% [19, 22–25], which is consistent with our findings. The same occurred for the results obtained with the ANSD, the incidence of which can vary from 0.1% to 4% [26–28]. The conductive hearing loss was the most frequent in this study. It must be analyzed and identified due to its significant presence and negative interference in language development of the child. Moreover, it can complicate the diagnosis of permanent hearing loss [13].

The most common etiology is otitis media, clinical entity characterized by acute inflammation of the mucosal lining of the middle ear. It is the most frequent diagnosis in children and becomes less common with advancing age. Children presented 65% of risk of developing an episode during the first 24 months. Otitis media is considered a highly prevalent disease in childhood, with higher peak incidence between 6 and 24 months of age, and the second peak incidence between 4 and 7 years old. Inflammation of the middle ear cavity results from the interaction of several factors. The most important factors are infection and auditory tube dysfunction, which result from immaturity of the immune system and also the structural and functional immaturity of the auditory tube [29]. Although otitis media is a floating condition that often affects one ear and usually causes mild hearing loss, it is clearly associated with major limitations in the development of language and speech. These children will be accompanied by professional staff, because it is already proven interference from conductive hearing loss in auditory and language development and future academic performance [30].

The cases of sensorineural hearing loss are in habilitation (hearing aids or cochlear implant) and speech rehabilitation process. Paludetti et al. [31] considered the rehabilitation as the most challenging management of sensorineural loss. The current procedures are represented by hearing aids and cochlear implants indication. However, recent advances in the basic area can represent the basis for new therapeutic procedures such as implantable device, brainstem implant, and cellular therapy.

The ANSD occurred in one neonate from GI, who was born at 38 weeks of gestational age and 3175 g of weight, remained 55 days in NICU, and presented the risk factors neonatal asphyxia and mechanical ventilation for prolonged periods, which were the probable etiology of the alteration. The infant refer NHS bilaterally on AABR to 45 dB and diagnosis showed no waves in the ABR, presence of cochlear microphonic and otoacoustic emissions, tympanometric curve type A, and no acoustic reflex. A study realized with temporal bones of preterm revealed 27% of selective loss of inner hair cells, higher prevalence than term infants (41% versus 28%) [32]. The infant was referred for indication and fitting of hearing aids and/or cochlear implants and speech therapy.

The results of NHS were analyzed, step test, considering the age in days after birth and postconceptual age at which screening was applied and the results passed or referred (Table 4). It was found that around 60% of children who passed the NHS in both GI and GII underwent a hearing screening within 30 days of life. A shorter residence in the neonatal intensive care unit assumed less severe cases, faster recovery of prenatal, perinatal and postnatal complications, and shorter use of specialized equipment and/or drug treatment. These conditions caused less damage to the auditory system and consequently more children passed the NHS. There were neonates who underwent a hearing screening with more than 30 days of life, due to a prolonged stay in the NICU.

None of the infant GI or GII with postconceptual age less than 34 weeks failed the NHS, so the postconceptual age did not influence the results of NHS. The literature recommended that NHS conducted through AABR be applied from 34 weeks so there is no influence of the maturity of the auditory system, especially up the brainstem, in the pass/fail result [33]. The findings of this study are consistent with the literature that shows that the NHS should be performed as soon as possible, considering only the clinical condition of the infant, and unnecessary delay of the NHS should be avoided [34].

Children who passed, but presented risk indicators for progressive and/or late hearing loss, were referred for hearing and language follow-ups at 6, 12, 18, and 24 months. This procedure also aimed to detect any upward or conductive losses that, given the characteristics of procedure adopted, passed the NHS. The literature indicated that the follow-ups conducted in infants in the NICU showed that hearing deteriorated in a small percentage of children [34]. It was diagnosed progressive or late onset hearing loss in children followed until the age of three with less than 28 weeks of gestational age and weighing less than 1250 g [35].

The dropout at various stages of NHS and audiological diagnosis was computed. No significant difference was found between evasion in different stages for GI and GII. Considering the screening, stage test, and diagnostic in GI, dropout was 21.7%. In the retest performed in GII, dropout was 2% and, considering the whole process, about 24% (Table 5). Therefore, dropout was not affected by retest applied in GII. In the study performed by Korres et al. [36], the retest decreased the false positive but increased evasion. In addition, to further reduce the evasion rate, it is necessary to implement a system for search and follow-up of cases, humanized assistance, and elaboration of educational materials to families.

The evasion of hearing care programs is a global reality. States with programs of early detection and intervention more developed in the United States include prevalence 2-3/1000 infants with hearing loss, but there are states with lower prevalence, probably due to dropout. In the United States, only 55.2% of children who failed the hearing screening attended diagnosis and 64.3% are in rehabilitation programs [16, 23]. Evasion for the diagnosis ranged from 1.73% to 81.25%. For the retest, dropout values ranged from 23.1% to 62% [24, 36, 37]. Reduced dropout rate of 1.73% [38] was obtained as a result of good communication between professionals who had made the screening and diagnosis; besides all children had received a letter with the failed result.

Adherence to hearing health programs is a challenge that must be overcome in order to achieve the goals of early detection of hearing loss. The low attendance at prenatal consultations (one to three visits), having more than one child in the family, the absence of a partner, in addition to low maternal education, lack or low number of available services, and lack of knowledge of responsible membership interfered in the adhesion in child health program [23]. Socioeconomic factors can have a significant influence on the effectiveness of NHS programs in low-income countries [14]. It was recommended that the prenatal consultation issues concerning the importance of hearing in child development and the real possibility of early detection of hearing loss should be addressed in order to introduce new elements for mothers' reflection about childhood hearing loss and their damage. The importance of the NHS should also be disseminated among health professionals, especially pediatricians, accompanying infants systematically during routine visits and could encourage the return to service until the conclusion of the TAN and reinforce the importance of early detection of hearing loss on child development.

The most common risk factors in the studied sample were NICU patients who stayed for more than 5 days, preterm birth, use of mechanical ventilation, being small for gestational age, exposure to ototoxic drugs, weight of  less than 1500 g, and Apgar score of 0–4 in the first minute (Table 6). These results were expected, since the sample consisted of newborns who required intensive care and remained at least 48 hours in NICU and/or intermediate care. In Bielecki et al. [39] study, ototoxic medication was the most common risk factor, followed by premature birth, low birth weight, and long admittance in ICU. Severe asphyxia and mechanical ventilation were more frequent in Hille et al. study [40].

It was found that craniofacial anomalies involving the ear and temporal bone significantly influenced the outcome of the hearing screening and audiological diagnosis. Neonates with this indicator showed a greater number of failures in the hearing screening, diagnosis of hearing loss compared to normal hearing, and conductive loss in relation to sensorineural.

We compared the results obtained in hearing screening and audiological diagnosis of neonates who had at least one indicator of the risk factors, including birth weight less than 1500 g, preterm birth, anoxia neonatal, and congenital infections with the results of neonates who presented the other studied risk factors. This analysis was conducted because they are often present in clinical histories of newborns that remained in the NICU. It has been found that these indicators did not influence the result of hearing screening. Comparing the diagnosis of normal hearing and hearing loss, there was a significant difference, with normal hearing the most frequent result. The statistical difference also occurred when comparing the type of hearing loss; the most frequent was conductive hearing loss.

Genetic syndromes that usually express hearing loss significantly influenced the result of  NHS, step test for GI and GII and retest stage for GII, resulting in higher failure rate in neonates with this factor. GI and GII preterm neonates showed a lower percentage of referral than the others in the test stage. The risk indicator neonatal asphyxia influenced the diagnosis. The only neonate with this indicator showed sensorineural hearing loss.

Comparing our results with the literature, it was verified that low birth weight, mechanical ventilation, and growth retardation were significantly associated with reference to NHS, unlike our findings [12]. Literature studies also found a significant relationship between hearing loss and the risk factors: neonatal asphyxia [23, 35], syndromes associated with hearing loss [23], and craniofacial anomalies [23]. Craniofacial anomalies had a significant relationship with conductive hearing loss [11], as in this study.

It is essential to consider the risk factors present in the clinical history of newborns, not to select candidates for hearing screening, but to serve as a guide to the most appropriate management for each case. It is imperative that infants with risk indicators that influenced the results of the hearing screening and diagnosis of hearing loss attend the necessary steps of the hearing child health program.

## 5. Conclusion

From the analysis of the results the following was concluded.

- It was not possible to perform universal neonatal hearing screening in the sample. The postconceptual age did not influence the result of hearing screening; therefore, it should not be unnecessarily delayed. It should be performed as soon as possible, only considering the clinical condition of the infant.
- Retesting reduced the failure rate, the rate of false positives and did not interfere with dropout, so it is a recommendable stage in ICU hearing screening programs.
- The incidence of hearing loss was 2.9%, considering sensorineural hearing loss, conductive hearing loss, and auditory neuropathy spectrum. The incidence of sensorineural hearing loss was 1.04%.
- Strategies to minimize the evasion in child hearing health programs should be implemented.
- Infants with craniofacial anomalies involving the ear and temporal bone showed a higher number of failures in the hearing screening of hearing loss diagnosis compared to normal hearing and of conductive loss in relation to sensorineural. The risk factors of genetic syndromes that often express hearing loss and prematurity had significant influence on the outcome of hearing screening.

## Figures and Tables

**Box 1:**
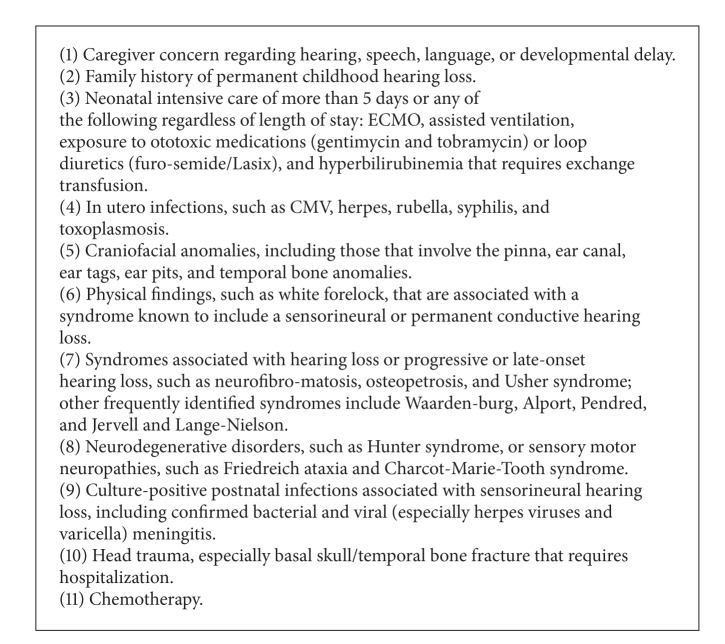
Risks indicators associated with hearing loss in childhood (JCIH, 2007) [5].

**Table 1:** Infants from the GI and GII, considering the results of the newborn hearing screening.

NHS	GI-test		GII-test and retest		*P* value*
	*n*	%	*n*	%	
Pass	337	81.4%	324	95.9%	**<0.0001**
Refer	77	18.6%	14	4.1%	

*Chi-square test/Fisher exact test.

**Table 2:** Infants from the GI and GII, considering the ENT and audiological diagnosis.

Diagnosis	Group I		Group II		Total		*P* value*
	*n*	%	*n*	%	*n*	%	
Normal	28	62.2%	1	12.5%	29	54.7%	**0.0098**
Conductive	9	20.0%	5	62.5%	14	26.4%	
Sensorineural	6	13.3%	1	12.5%	7	13.2%	
ANSD**	1	2.2%	0	0%	1	1.9%	
Death	1	2.2%	1	12.5%	2	3.8%	

Total	45	100%	8	100%	53	100	

**ANSD: auditory neuropathy spectrum disorder/*Fisher exact test.

**Table 3:** Infants from GI and GII considering the incidence of hearing loss.

Hearing loss	Group I		Group II		Total	
	*n*	%	*n*	%	*n*	%
Conductive	9/414	2.2%	5/349	1.4%	14/763	1.8%
Sensorineural	6/414	1.44%	1	0.3%	7/763	0.91%
ANSD	1/414	0.24%	0	0%	1/763	0.13%

Total	16/414	3.9%	6	1.7%	22/763	2.9%

**Table 4:** Infants from the GI and GII, which held the NHS first test considering the time in days after birth and postconceptual age, in weeks, in which hearing screening was applied and the result passed and failed.

Hearing screening	GI					GII				
	Referral		Pass		*P* value	Referral		Pass		*P* value
	*n*	%	*n*	%		*n*	%	*n*	%	
Time after birth (days)					0.1805					**<0.0001**
<30	49	63.6	198	59.1		28	59.6	187	65.6	
≥30 a < 60	10	13.0	74	22.1		0	0.0	52	18.2	
≥60	18	23.4	63	18.8		19	40.4	46	16.1	
Postconceptual age					0.1713*					0.7343*
<30	0	0.0	0	0.0		0	0.0	1	0.4	
≤30 a < 34	0	0.0	1	0.3		0	0.0	8	2.8	
≤34 a < 37	19	24.7	92	27.5		9	19.1	95	33.3	
≥37	58	75.3	242	72.2		38	80.9	181	63.5	

*Chi-square test/Fisher exact test.

**Table 5:** Infants from the GI and GII, considering the dropout rate in the various stages of NHS and audiological diagnosis.

Stages	Group I		Group II		*P* value*
	*n*	%	*n*	%	
NHS-first stage	74/488	15.2%	92/441	20.9%	0.4012
NHS-retest	—	—	9/441	2.04%	
Diagnosis	32/488	6.55%	6/441	1.360%	

Total	106/488	21.7%	106/441	24.03%	

*Chi-square test.

**Table 6:** Infants who underwent hearing screening, step test, considering the frequency of each risk (*n* = 763).

Risk factors	*n*	%
Family history of permanent childhood hearing loss	7	0.9
Birth weight less than 1500 g	153	20.1
Preterm birth	508	66.8
Neonatal intensive care of more than 5 days	537	70.6
Assisted ventilation	279	36.7
Exposure to ototoxic medications or loop diuretics	239	31.4
Hyperbilirubinemia that requires exchange transfusion	42	5.5
Craniofacial anomalies	22	2.9
Severe perinatal anoxia	44	5.8
In utero infections (CMV, herpes, rubella, syphilis, and toxoplasmosis)	43	5.7
Syndromes associated with hearing loss	15	2.0
Neurodegenerative disorders	3	0.4
Culture-positive postnatal infections associated with sensorineural hearing loss	7	0.9
Head trauma	2	0.3
Chemotherapy	1	0.1
